# Deep-Learning-Based Cryptanalysis of Lightweight Block Ciphers Revisited

**DOI:** 10.3390/e25070986

**Published:** 2023-06-28

**Authors:** Hyunji Kim, Sejin Lim, Yeajun Kang, Wonwoong Kim, Dukyoung Kim, Seyoung Yoon, Hwajeong Seo

**Affiliations:** Department of Convergence Security, Hansung University, Seoul 02876, Republic of Korea; 22111401@hansung.ac.kr (H.K.); 22213202@hansung.ac.kr (Y.K.);

**Keywords:** cryptanalysis, deep learning, lightweight block ciphers, S-DES, S-AES, S-SPECK

## Abstract

With the development of artificial intelligence, deep-learning-based cryptanalysis has been actively studied. There are many cryptanalysis techniques. Among them, cryptanalysis was performed to recover the secret key used for cryptography encryption using known plaintext. In this paper, we propose a cryptanalysis method based on state-of-art deep learning technologies (e.g., residual connections and gated linear units) for lightweight block ciphers (e.g., S-DES, S-AES, and S-SPECK). The number of parameters required for training is significantly reduced by 93.16%, and the average of bit accuracy probability increased by about 5.3% compared with previous the-state-of-art work. In addition, cryptanalysis for S-AES and S-SPECK was possible with up to 12-bit and 6-bit keys, respectively. Through this experiment, we confirmed that the-state-of-art deep-learning-based key recovery techniques for modern cryptography algorithms with the full round and the full key are practically infeasible.

## 1. Introduction

Cryptanalysis for block ciphers has been studied and is still receiving high attention. There are various methods, such as linear cryptanalysis [1], differential cryptanalysis [2], and side-channel analysis [3]. The linear attack is a known-plaintext attack, and it is a method of finding a key by linearizing the non-linear structure inside the cryptographic algorithm. Differential analysis is an attack technique that can be used in chosen-plaintext attacks. In addition, there are various deep-learning-based cryptanalysis [4,5,6] based on advanced computing power and quantum-computer-based cryptanalysis (e.g., Shor’s algorithm [7] and Grover’s algorithm [8]).

In this paper, we present deep-learning-based cryptanalysis for lightweight block ciphers including simplified DES (S-DES) [9], simplified AES (S-AES) [10], and simplified SPECK (S-SPECK) [11] (it is a simplified version of DES [12], AES [13], and SPECK [11] ciphers). In the case of cryptanalysis for S-DES, it was first performed in [6], but it still has room to improve with the state-of-art deep learning techniques. We performed the attack with fewer parameters ensuring higher accuracy by applying the-state-of-art deep learning technology. Furthermore, to the best of our knowledge, this work is the first key recovery attack based on deep learning for S-AES and S-SPECK. Significantly, our results demonstrate a partial (round-reduced and key-reduced versions) cryptanalysis using deep learning, and it indicates that current deep-learning-based cryptanalysis is not feasible in practice.

### 1.1. Contribution

The following are the major contributions of this work.

#### 1.1.1. Designing Artificial Neural Networks Considering the Characteristics of Cryptographic Algorithms

In order to design an optimal artificial neural network for cryptanalysis, we constructed a neural network considering the characteristics of the cryptographic algorithm. Various types and options of neural networks were tested, and we selected the neural network structure with the best performance.

#### 1.1.2. Improving Performance for S-DES Cryptanalysis

We applied the-state-of-art artificial neural network techniques that were not applied in the previous work for deep-learning-based cryptanalysis. The neural network implemented in our work achieved 5.3% higher accuracy with 93.16% fewer parameters compared to previous work in cryptanalysis for S-DES.

#### 1.1.3. The First Key Recovery (Random Bit Key) Based on Deep Learning for S-AES and S-SPECK

We are the first to attempt the deep-learning-based key recovery (for random-bit key) against S-AES and S-SPECK, to the best of our knowledge. We confirmed that cryptanalysis is possible for S-AES and S-SPECK up to 12-bit and 6-bit key spaces. Finally, we compared the results of cryptanalysis for S-DES, S-AES, and S-SPECK.

### 1.2. Organization

The remainder of this paper is organized as follows. In Section 2, related technologies, such as artificial neural networks, deep-learning-based cryptanalysis, and previous work, are presented. In Section 3, the proposed cryptanalysis based on an artificial neural network is introduced. In Section 4, the evaluation of our deep-learning-based advanced cryptanalysis technique implementation is discussed. Finally, Section 5 concludes the paper.

## 2. Preliminaries

### 2.1. Artificial Neural Network

**Figure 1:**
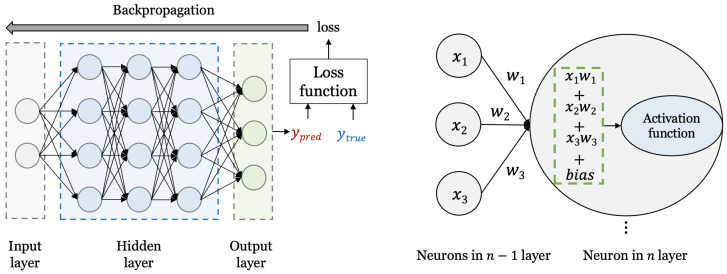
Architecture of artificial neural networks.

Artificial neural networks [14] are learning algorithms inspired by neural networks in biology. As shown in Figure 1, a neural network is constructed in the form of stacked layers of multiple nodes. As shown on the right of Figure 1, neurons (i.e., nodes) in each layer perform a multiplication and sum operation using the node values (*x*) and weights (*w*) of the previous layer connected to them and add a bias [15]. Then, it is input to the non-linear activation function [16] and computed as a single value. In this way, the loss value is obtained after passing through all the layers. Then, the weights inside the neural network are updated to minimize the loss through the backpropagation process. By repeating this process, a neural network that guarantees generalization performance for untrained data is constructed. When the trained model is used for actual inference, the inference proceeds by inputting data with the weights of the fixed neural network. Through this, it is possible to learn, classify, and predict by extracting features of input data (e.g., image, time series, language, and graph). The type of neural network is appropriately selected according to the characteristics of the input data. There is the most basic network, which is multi-layer perceptron (MLP); the convolutional neural network (CNN) [17], which is good for image learning; and the recurrent neural network (RNN) [18], which is effective for time series data prediction. There are also generative adversarial networks (GANs) [19], which are used for tasks that generate data, and techniques such as reinforcement learning [20], which allows the optimal action to be selected based on the concept of a reward. A loss function and an optimization function are used in the training process for an artificial neural network. The loss function calculates the loss, which is the difference between the actual answer and the predicted value. Loss functions include binary cross-entropy, categorical cross-entropy, mean squared error, etc., and are selected according to the problem to be solved (e.g., binary cross-entropy is used for binary classification). The neural network needs to minimize the loss calculated in this way, and an optimization function is used for this. Optimizers [21] (e.g., stochastic gradient descent (SGD), RMSprop, and Adam) are used to effectively find the minimum.

#### 2.1.1. Residual Connections in Neural Networks

**Figure 2:**
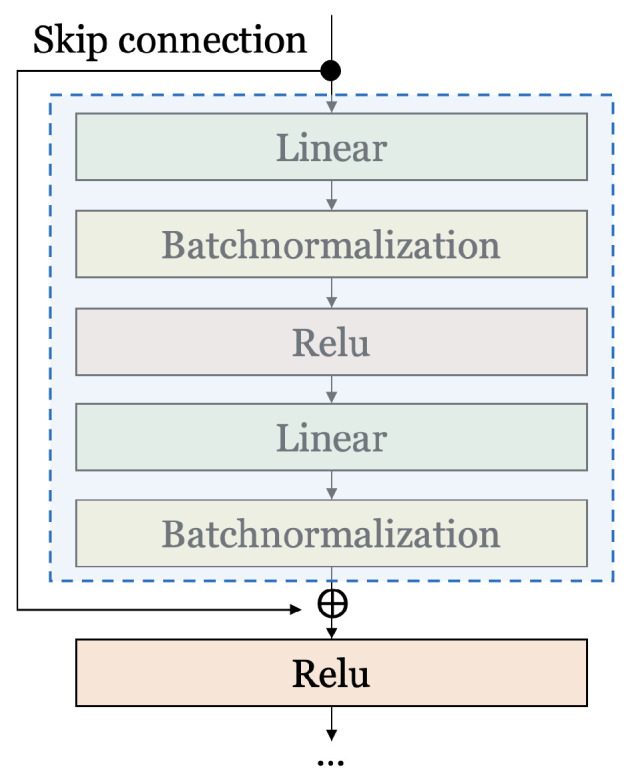
Skip connection for the residual network.

Figure 2 shows residual connections (i.e., skip connections) [22] in an artificial neural network. As shown in Figure 2, a residual connection has a structure in which the output of the previous layer is added to the input of the next layer after skipping several layers. This structure allows our data to go deeper into the neural network by following skip connections instead of following the main path where data flow by default. In other words, the skip connection means skipping layers to propagate information to a deeper layer. If the network gets deeper, the gradient may vanish as the gradient converges to 0 as it goes to the input layer in the backpropagation process. The residual connection can solve this gradient vanishing problem. Larger gradients can be propagated to the initial layer, and the initial layers are trained as fast as the output layer. This structure allows deeper networks to be trained. Additional information can be learned and the overfitting of neural networks can be prevented.

#### 2.1.2. Gated Linear Units

**Figure 3:**
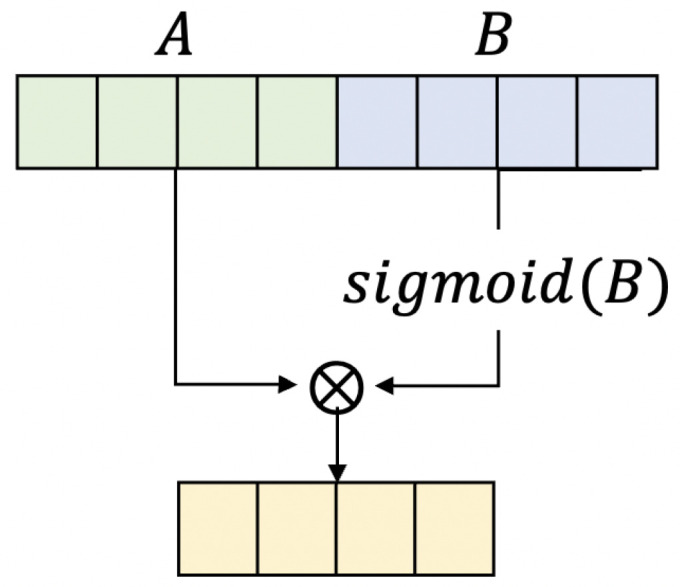
Gated Linear Unit (GLU).

Figure 3 shows a gated linear unit (GLU) [23]. GLUs control the input data like a gate. In each layer, a matrix multiplication operation of input data and weights is performed, and then it is input to the activation function. In the case of GLUs, *A* and *B* are constructed, as shown in Figure 3, before input to the activation function. After that, the *B* part is input to the sigmoid activation function. Since the sigmoid function is a bipolar function, important data survives in the multiplication process. Conversely, insignificant data with generally small values have smaller values. Finally, a point-wise multiplication on *A* and *B* is performed. In other words, the GLU allows us to focus on more important information and enables faster and more stable training.

### 2.2. Artificial Neural Network-Based Cryptanalysis

The development of artificial intelligence technology could provide a new approach for cryptanalysis [24]. In the artificial-neural-network-based cryptanalysis, known-plaintext attacks, ciphertext-only attacks, and differential attacks against Caesar cipher, Vigenere cipher, simplified-DES, round-reduced SPECK, and round-reduced SIMON were mainly performed [4,5,6,25,26]. In addition, the work of predicting the number of active S-boxes required for cryptanalysis was also studied [27]. There are mainly two types of cryptanalysis using artificial neural networks (i.e., known-plaintext attack and differential attack). For known-plaintext attacks, S-DES, SPECK, and SIMON block cipher algorithms are targeted [6]. They performed cryptanalysis on text-based keys and random-bit keys. However, in both cases, only S-DES could be attacked, and SPECK and SIMON could only analyze text-based keys. In [28], a known-plaintext attack on Caesar cipher was performed using quantum support vector machine (QSVM) [29] through quantum machine learning. QSVM has a feature map of the existing support vector machine (SVM) [30] designed as a quantum circuit on a quantum computer, and like SVM, it is a machine learning technique that finds the optimal boundary between data points through a hyperplane. They performed cryptanalysis for 2-bit and 3-bit plaintext and ciphertext pairs and keys as resource and memory problems such as qubits required for the quantum circuits.

### 2.3. Previous Work

As noted earlier, known-plaintext attacks for S-DES, SPECK, and SIMON have been proposed in [6]. In this approach, plaintext and ciphertext pairs are expressed as bits, concatenated, and then input into a neural network. Then, the neural network predicts the key corresponding to the pair of plaintext and ciphertext by comparing it with the real key. Finally, it calculates the loss through the MSE (mean squared error) loss function (https://www.tensorflow.org/api_docs/python/tf/keras/losses/MeanSquaredError (accessed on 25 June 2023)). Training is carried out to minimize the loss to predict the correct key corresponding to the pair of plaintext and ciphertext. The weights of the neural network trained to achieve sufficient performance through this process are fixed. In the inference phase, the key can be predicted using a trained neural network. The number of pairs of plaintext and ciphertext used for training and validation of S-DES are 50,000 and 10,000, respectively. The number of pairs of plaintext and ciphertext used in SPECK and SIMON’s training and validation are 500,000 and 1 million. In their experiment, they used the random-bit key and text key. The random-bit key has the same probability of occurrence of all bits, the key space for *n*-bit is $2^{n}$, and the text-based key uses only 64 ASCII codes. The probability of occurrence of all bits is not the same. That is, the text key is easier to predict because text-based keys have a different probability of occurrence for each bit, and the key space is smaller than for random-bit keys. Bit accuracy probability (BAP) and deviation were used to evaluate the performance of the previous work. If the probability of occurrence for each bit is different, it is easier to predict. For example, if the first bit is 1 with a probability of 1.0, the neural network can predict the first bit as 1 without difficulty. In other words, the difference between the BAP and the probability of occurrence is calculated to fairly evaluate the performance when the probability of occurrence of the key for each bit is different. The difference between the two values is calculated, and if the value is a positive number, it is determined that cryptanalysis is possible. In the case of the random-bit key, since the key has the same probability of occurrence for all bits, the deviation is the value obtained by subtracting 0.5 from BAP.

As a result of the experiment, in the case of S-DES, cryptanalysis was possible for both the random-bit key and the text key. In addition, the fifth and eighth bits in the random-bit key and the text key were vulnerable to attack, and the sixth bit was relatively safe in cryptanalysis. Next, SPECK and SIMON achieved an average prediction probability of 0.5 for the case of using a random-bit key, and there were bits with a negative deviation. Cryptanalysis failed for random-bit keys and succeeded for text keys (keyspace is $2^{48}$ and key occurrences are different).

In our work, the performance of cryptanalysis is improved by applying the-state-of-art artificial neural network structure and technology with well-selected parameters.

### 2.4. Target Lightweight Cipher

A lightweight cipher is an encryption method characterized by small space and/or low computational complexity [31]. The lightweight ciphers we target in our work are S-DES, S-AES, and S-SPECK. The details of each cipher are as follows.

- S-DES: It is a simplified version of the DES. It has an 8-bit block size and a 10-bit key size. S-DES consists of initial permutation (IP), Cipher function *f* (expansion, key addition, substitution, and permutation), and swap function. The encryption process can be demonstrated as $C=E\left(P,K\right)=IP^{-1}(\rho_{2}\left(\rho_{1}\left(IP\left(P\right)\right)\right)$. Before the 1-round, the plaintext that has been subjected to initial permutation is divided into $L_{0}$ and $R_{0}$ (these are plaintext). The round function (ρ) is performed twice and is calculated as: $L_{i}=R_{i-1}$, $R_{i}=L_{i-1}⊕f\left(R_{i-1},K_{i}\right)$. In key scheduling, two 8-bit subkeys are generated by permutation and shift operations on a 10-bit key. Therefore, it has a 10-bit master key and two 8-bit subkeys.
- S-AES: It consists of nibbles substitution (NS), shift rows (SR), mix columns (MC), and key addition ($A_{K}$). The key expansion and encryption are based on an S-box (94ABD1856203CEF7) that depends on a finite field with 16 elements. It has a 16-bit key, operates on 16-bit plaintext, and has two rounds. The encryption consists of 8 functions ($A_{K_{2}}\circ SR\circ NS\circ A_{K_{1}}\circ MC\circ SR\circ NS\circ A_{K_{0}}$) for two round, and $A_{K_{0}}$ is applied first.
- S-SPECK: S-SPECK used in our work does not mean a simplified structure (function) but an implementation with reduced key space and reduced rounds. It has 10 variants, but we consider only 32-bit plaintext here. It consists of a 128-bit key and 22 rounds in full version. However, we start cryptanalysis with one round and 1-bit key space, extending it as much as possible (if we succeed in analyzing the full round and full key, our target is not S-SPECK). S-SPECK has the same structure as SPECK. So the round function operates like this. First, the round function divides the input value into *l* and *r*, and rotation, addition, and xor are performed as follows: $l_{i}=\left({\mathrm{ROR}}_{7}\left(l_{i}\right)⊞r_{i}\right)⊕k_{i}$ and $r_{i}={\mathrm{ROL}}_{3}\left(r_{i}\right)⊕l_{i}.$

## 3. Deep-Learning-Based Key Recovery for Lightweight Block Ciphers

**Figure 4:**
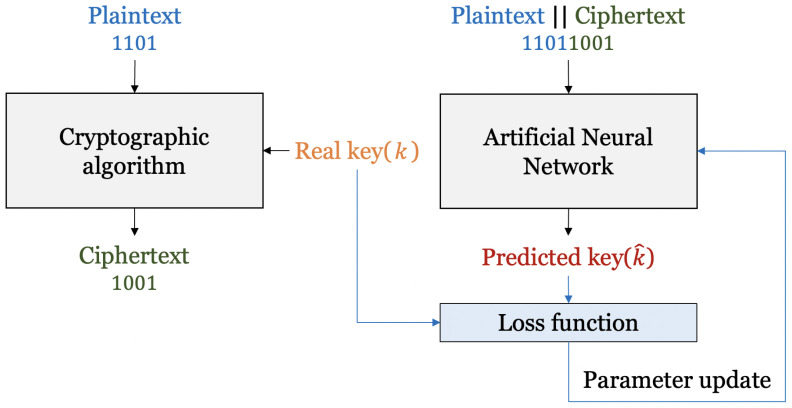
System diagram for the proposed method.

In this paper, we propose an advanced cryptanalysis technique based on deep learning for S-DES. Several deep learning technologies that can improve performance compared to previous work were applied. We also try cryptanalysis for S-AES and S-SPECK with the random-bit key. Figure 4 shows the system diagram for the proposed method. First, a cryptographic algorithm is used to obtain a pair of ciphertexts and plaintext. The real key used is called *k*. The real key *k* is used as a label for the data to be trained on. Next, we concatenate plaintext and ciphertext, which are then inputted to an artificial neural network for supervised learning. The neural network learns the characteristics of the input data. It can predict the correct label. The output of the neural network is $\hat{k}$, which is the predicted key, and it is input to the loss function to compare with the real key *k*. As the real key and the predicted key become similar, the loss becomes minimized, and the neural network updates the weight to minimize the loss. The neural network is trained by repeating this process to make correct predictions.

### 3.1. Data Generation

**Figure 5:**
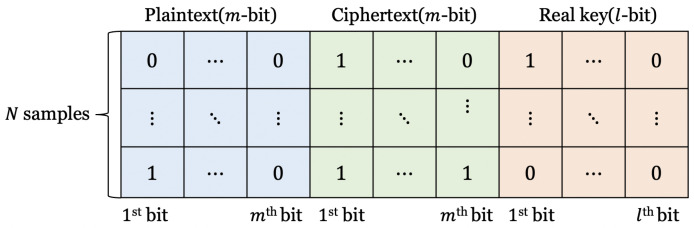
Dataset.

Figure 5 shows the data set for training and test, and Table 1 shows details of the data set for S-DES [9], S-AES [10], and S-SPECK [11]. The data type is bits. Plaintext and ciphertext pairs and keys are expressed as bits. When saving these as a CSV file format for training, one bit is inputted per one column. When the length of the plaintext is *m*, the data have a length of 2m, and when input to the neural network, one bit is assigned to each neuron. The key bit of *l*-bit is used as a label. In other words, it is not a classifier classified as a value from 0 to $2^{l}-1$ but predicting each bit of the key.

We sampled plaintext and keys randomly. In the case of S-DES, an 8-bit block and a 10-bit key are randomly selected and then encrypted to make a data set. S-AES randomly samples 16-bit plaintext, and the 16-bit key then encrypts it to form a data set. S-SPECK has 32-bit plaintext and a 64-bit key. They are randomly chosen. The probability of occurrence of all bits is the same because we generate the random number in the range of $2^{n}$. Let the number of data set for training be $N_{tr}$, the number of data set for validation be $N_{val}$, and the number of data sets for testing be $N_{ts}$. In addition, S-AES and S-SPECK have a longer plaintext and a longer key length than S-DES. So, they require a large number of data.

### 3.2. Neural Network Structure for Cryptanalysis

There are various types of neural networks that can be used for cryptanalysis, such as MLP, CNN, and RNN. Among them, we use a fully connected neural network to design an effective artificial neural network for cryptanalysis. The reason has to do with the properties of a cryptographic algorithm. A cryptographic algorithm has a property that most or all bits are affected when a single bit is changed, and half of the ciphertext is statistically changed when a single bit of the plaintext is changed [32]. In other words, the first bit of the plaintext can affect all bits of the ciphertext. Therefore, it is difficult for data for learning to have a locality in which adjacent features have similar values, and it is not temporal data having time information. Therefore, instead of CNN and RNN, which are effective for training data with temporal locality, a fully connected network suitable for considering global information of data is used.

#### 3.2.1. Structure of Neural Network for Cryptanalysis for S-DES

**Figure 6:**
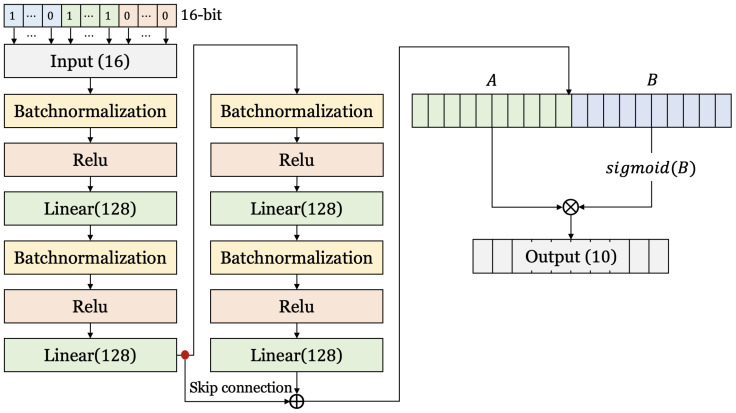
The neural network structure for cryptanalysis for S-DES.

Figure 6 is the neural network structure for S-DES cryptanalysis. We designed a neural network by applying the residual connection and GLU described above. Input data are 16-bit because each bit of the data set concatenated 8-bit plaintext and 8-bit ciphertext. The number of neurons in the input layer was set to 16. That is, each bit is input to each neuron. Additionally, the same number of neurons was used in all hidden layers to minimize information loss. Finally, it goes through the GLU, which enables stable learning by controlling the information. The number of neurons in the output layer is 10, equal to the number of key bits. That is, each neuron in the output layer predicts each bit of the key.

#### 3.2.2. Structure of Neural Network for Cryptanalysis for S-AES

**Figure 7:**
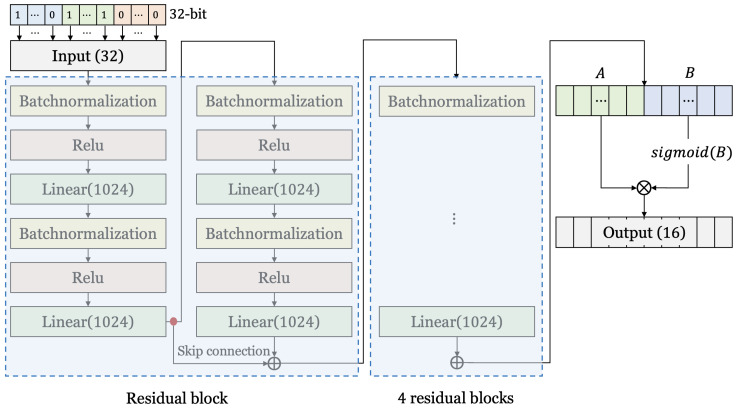
The neural network structure for cryptanalysis for S-AES.

Figure 7 shows the structure of the neural network for cryptanalysis for S-AES. For S-AES, we set the number of input neurons to be the same as the number of bits of input data in the same way as S-DES. In addition, five residual blocks and one GLU are applied, and the number of neurons in each hidden layer is larger than that of S-DES. In other words, it has a structure similar to the neural network used for S-DES, but it uses a deeper and larger neural network.

#### 3.2.3. Structure of Neural Network for Cryptanalysis for S-SPECK

**Figure 8:**
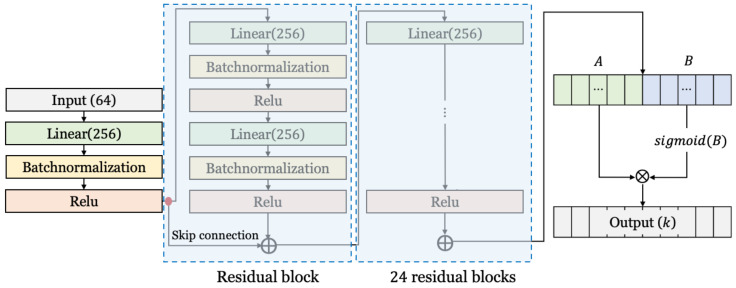
The neural network structure for cryptanalysis for S-SPECK.

Figure 8 shows the architecture of the neural network for key recovery attack for S-SPECK. The model for S-SPECK is much deeper and larger than the neural networks for S-DES and S-AES. The hidden layer consists of 25 residual blocks (including linear, batch normalization, and Relu). The units of the linear layer of each block are 256. Unlike the previous two models, the output layer has *k* neurons. Since the key length of SPECK is 64, the number of output units should originally be 64, but then the size of the model becomes too large. So, the number of output neurons was reduced to predict only the reduced key bits.

### 3.3. Training and Testing

#### 3.3.1. Training

Training and testing are performed using a neural network designed for cryptanalysis and the prepared data. First, for training, a training data set is input to a neural network, and the neural network outputs predicted values. The output value and the real key are input to the loss function, and the loss represents the difference between the two values (predicted value (${\hat{k}}_{i,j}$) of the *j*-th bit of the *i*-th, and the real key (*k*)) is calculated. In our work, MSE is used to calculate the loss between the real key and the predicted key and is shown in Equation (1). *N* is the number of data samples ($N_{tr}$ or $N_{val}$), and *L* is the number of key bits. In order to minimize the loss, the training is performed while repeating the process of updating the weights of the neural network. If the training is not performed properly, the neural network will output a predicted value of about 0.5 or an incorrect value. If the training is performed properly, the neural network will predict the output values similar to the real key value.

(1) $$\begin{array}{c} \frac{1}{N\cdot L}\cdot \sum_{i=1}^{N}\sum_{l=1}^{L}{\left({\hat{k}}_{i,l}-k_{i,l}\right)}^{2} \end{array}$$

#### 3.3.2. Testing

In the inference phase, a trained neural network that has fixed weights is used. This neural network outputs predicted values ($\hat{k}$) when inputting test data. This value is a real number. We need to compare $\hat{k}$ with the real key (bit) for the test. In other words, $\hat{k}$ must be converted to 0 or 1, because it needs to be compared in the form of a bitstring. For this, the predicted key value ($k_{pred(i,l)}$) is calculated as in Equation (2) using the predicted value (${\hat{k}}_{i,l}$) of the *l*-th bit of the *i*-th data sample in the test data set. In addition, *BAP* (the accuracy of each key bit for the entire data set) is calculated using the transformed predicted key and the real key as in Equation (3). If the training is poorly performed, BAP is less than 0.5. That is, the neural network just guesses the result with one of two. With the proper training, the BAP will be greater than or equal to 0.5. If the result is 0.5 or greater, the neural network can predict the corresponding key bit.

(2) $$k_{pred(i,l)}=\left\{\begin{array}{cc} 0 & {\hat{k}}_{i,l}<0.5,\\ 1 & {\hat{k}}_{i,l}\geq 0.5. \end{array}\right.$$

(3) $$\begin{array}{c} BAP_{l}=\frac{1}{N_{ts}}\cdot \sum_{i=1}^{N_{ts}}XNOR(k_{pred(i,l)},k_{\left(i,l\right)}) \end{array}$$

## 4. Evaluation

### 4.1. Experiment Environment

For this experiment, Google Co-laboratory PRO PLUS (commercial license), a cloud-based service, is utilized. It runs on Ubuntu 18.04.5 LTS and consists of an Nvidia GPU (Tesla T4) with 50 GB RAM. In terms of the programming environment, Python 3.7.13, TensorFlow 2.8.0 and Keras 2.8.0 version are used. Due to a large amount of data and the growing size of the neural network, it takes about 10 h to train one time.

### 4.2. Experiments on S-DES

#### 4.2.1. Training Result

We perform training on S-DES using the data and the structure of the neural network proposed in Section 3. We only show the results on our best neural network model. The loss function, optimizer, and epoch used for training are as follows.

- **Epoch:** As a result of 100 epochs in the network that both techniques are applied, the loss was sufficiently reduced (this means that the loss is no longer decreasing). The network that applies the skip connection requires 150 epochs. In the case of the previous work, 5000 epochs are performed. Since the GLU and residual connection technology are added to this work, stable and fast training is possible compared to the basic network.
- **Loss:** We used the MSE loss function. As a result of training a neural network to which a residual connection is applied, the training loss is 0.1656 and the validation loss achieves 0.1660. The results of training a neural network to both the residual connection and GLU are applied. The training loss achieves 0.1774 and the validation loss achieves 0.1767.
- **Optimizer:** We use the Adam optimizer. The optimizer is a function that finds the minimum value of the loss function (to minimize the loss). When the optimizer moves toward the minimum, its stride is called the learning rate. The learning rate of the optimizer is set as the learning rate exponential decay method. The range of the learning rate is from 0.001 to 0.1. Learning rate decay uses the large learning rate value at first, and then the value gradually decreases. This allows the neural network to achieve optimal training results faster.

These results show that the neural network to which GLU is applied can converge more stably and faster than when it is not applied.

#### 4.2.2. Bit Accuracy Probability

We perform inference using a trained neural network. Table 2 shows the result of inference for cryptanalysis. For comparison of the number of parameters with the previous work, the neural network described in [6] was implemented identically, and a similar performance was obtained. However, for the BAP excluding the number of parameters, the values written in the paper are used.

The seventh bit achieves less than 60% accuracy. They are relatively safe for cryptanalysis. However, the 6th, 9th, and 10th bits exceed 80% in accuracy. These bits are vulnerable to cryptanalysis attacks. A similar pattern to ours can be seen in previous work as well. Our method with both residual connection and GLU achieved higher overall accuracy. As a result of calculating the average over all bits, the accuracy is 5.3% higher. A slightly higher BAP is achieved than in previous works when the only residual connection is applied, and the BAP for the fourth bit exceeded 60%. Therefore, we can see that the neural network to which both techniques are applied is more effective for cryptanalysis.

Finally, with our neural network applying both techniques, we reduce the number of parameters by 93.16% compared to the previous work. As with the neural networks in the previous work, all layers of the neural network are fully connected layers. However, these are the results obtained by reducing the number of neurons in each layer from 512 to 128 and applying the residual connection and GLU. In other words, by applying this new deep learning technique, it is possible to achieve higher accuracy with a smaller neural network.

#### 4.2.3. Bit Accuracy Probability by Epoch

Table 3 shows the BAP by epoch. In every epoch, bits 4 and 7 are safe. This means that even if more training is performed, the accuracy will be low, so the predictions will be rather difficult. In addition, since the sixth and ninth bits in all epochs are vulnerable bits, they can be easily inferred even with little training. Additionally, as the epoch increases, the number of safe bits is decreased. That is, as the training progresses further, the accuracy of them increases so that they are no longer safe bits. The first and second bits are dropped from the safe bit from 20 epochs. Finally, after training with 100 epochs, the 10th bit, which is not vulnerable, is detected as a vulnerable bit. This result shows that the sixth and ninth bits are the most vulnerable, and the fourth and seventh bits are the most secure.

### 4.3. Experiments on S-AES

#### 4.3.1. Training Result

S-AES data are trained using the neural network structure proposed in Section 3. The loss function, optimizer, and epoch are as follows.

- **Parameters**: Cryptanalysis for S-AES requires much larger parameters than S-DES. The number of parameters for S-AES with 12-bit key space is 5,326,944, and the number of parameters for S-AES with 12-bit key space is 11,636,832. When the key space is increased by one bit, the number of parameters increases significantly compared to S-DES.
- **Loss**: In S-AES, the MSE loss function is also used. As a result of training, a training loss of 0.1826 and a validation loss of 0.1923 were achieved.
- **Optimizer**: We use the Adam optimizer [21], and it is also set to the learning rate exponential decay method (the range of learning rate is 0.001 to 0.1).
- **Epoch**: The epoch was set to 150. There is no decrease in loss even after training more than 150 epochs.

#### 4.3.2. Bit Accuracy Probability

Table 4 shows the result of cryptanalysis for S-AES. We train from 9-bit to 12-bit key spaces. For proper training, the capacity of the neural network must be large enough according to the complexity of the data. In the case of the 12-bit key space, even if the network is scaled as large as possible to accommodate the increased data complexity, training is hardly performed. In addition, training on very large neural networks and a large data set was not possible due to the constraints of the environment. This shows that deep-learning-based cryptanalysis requires a large data set, GPU, and memory environments. Through this experiment, we show that cryptanalysis of S-AES is possible up to an 11-bit key space. In the case of S-AES, unlike S-DES, the BAP of all bits is a similar value. When the key is increased by 1 bit, the accuracy tends to decrease by about 10% even if the capacity of the network increases.

### 4.4. Experiments on S-SPECK

#### 4.4.1. Training Result

In S-SPECK, the loss function, optimizer, and epoch are as follows.

- **Parameters**: The number of parameters of our model is 3,333,894. When a network smaller than this was used, there were cases where the BAP was less than 0.5. Additionally, since the size of the data set is large, out-of-memory (OOM) occurs. Therefore, the size of the model cannot be increased any further.
- **Loss**: We use the MSE loss function. The loss decreases to 0.2499. This result shows that it is barely trained considering the initial loss of 0.2548.
- **Optimizer**: We use the Adam optimizer with a cyclic learning rate (the range of learning rate is 0.001 to 0.002).
- **Epoch**: We set the number of epochs to 20. In order to the limitations of the experiment environment, we cannot use larger neural networks. Therefore, the accuracy did not improve even after learning more than 20 epochs.

#### 4.4.2. Bit Accuracy Probability

Table 5 shows the result of cryptanalysis for S-SPECK. In this experiment, more than 10,000,000 data are used, and to analyze more than a 6-bit key, more data are required. However, we can not use more data because of environmental constraints. To overcome this, we try different methods (increasing the capacity of the neural network and trying several methods to solve underfitting). However, analysis is not possible because the number of data is too small compared to the complexity of the data. In previous work, key recovery was possible because certain bits were determined with high probability when the text-based key was used. However, they failed analysis when they use random key bits. As shown by our results and previous work, it is difficult to infer a key when all key bits have a random probability. Therefore, if these resource and time limitations are not addressed, deep-learning-based key recovery is inevitably difficult.

### 4.5. Comparison of Cryptanalysis for S-DES, S-AES, and S-SPECK

**Figure 9:**
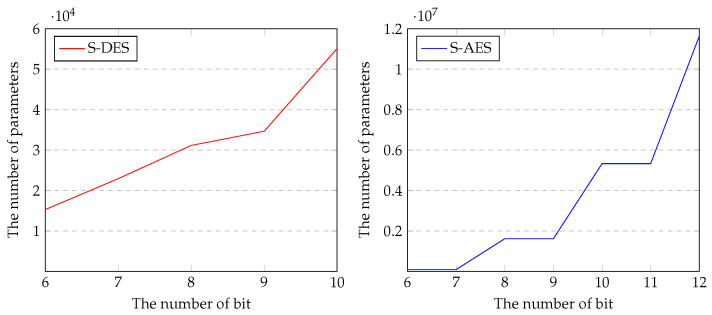
Parameters of neural network for cryptanalysis (**left**: S-DES, **right**: S-AES).

We obtained the following results through the experiments described above. First, S-DES has a specific vulnerability pattern compared to S-AES. In addition, S-AES has similar accuracy for all bits. As shown in Figure 9, the number of parameters required for cryptanalysis for S-AES is much larger and increases significantly. In general, the more complex data (e.g., larger dimensions, complex relationships between data features) need to be trained, the more capable the network is. Therefore, as the size of the plaintext of the target cipher increases and the security level increases, we use a larger neural network. In addition, in S-DES, when the key space increases by 1 bit, the number of parameters increases by about 1.5 times, but in the case of S-AES, it increases by about 2 to 3 times.

Considering that the accuracy is lower and the network requires much more parameters than that of S-DES when using the same key space as S-DES, it can be seen that S-AES is more difficult for neural networks to learn and is designed to be more secure than S-DES. S-DES consists of initial permutation, expansion/permutation, key addition, s-box (i.e., substitution), and swap operations. In addition, S-AES is composed of substitution nibbles, shift rows, mix columns, and key addition, and the key space is larger than that of S-DES. This result seems to have been derived because the S-box of S-AES is designed to be more confused than S-DES, so the relationship between the key and the ciphertext is not well revealed, and diffusion is better achieved through mix columns and permutation. That is, S-DES uses less key space and has poorer diffusion and confusion characteristics than S-AES. Some key bits can be inferred.

These results also apply to S-SPECK. It has a larger block size and key size than S-DES and S-AES, and it is designed with a much more complex structure. Therefore, even attacks on small key bits are not easy. That is, the larger the block and key size and the more complex the structure, the more difficult it is to infer the key using plaintext and ciphertext.

Finally, many parameters are required for cryptanalysis of the simplified cipher as shown in Figure 9, and many resources are required for training. Therefore, there is a limit to analyzing cryptographic algorithms such as AES with the current deep-learning-based technology, and it can be seen that text-key-based cryptanalysis is possible as in the previous work.

## 5. Conclusions

In this paper, we present an improved deep-learning-based cryptanalysis for S-DES, S-AES, and S-SPECK. In our improved neural network, a skip connection and gated linear units are applied for more stable learning. As a result, 5.3% higher accuracy was achieved on average, and the number of parameters was reduced by 93.16% compared to previous work in S-DES. We also performed deep-learning-based cryptanalysis for S-AES and S-SPECK. Key recovery is possible up to a 12-bit key for S-AES and up to a 6-bit key space for S-SPECK. Through two experiments, we can confirm that it is practically impossible to recover the entire key for a modern cipher (cryptanalysis of full rounds and full keys is very difficult due to the limitations of the current deep-learning approach (e.g., high computation and memory usage)). In future work, we will apply deep-learning-based cryptanalysis for other block ciphers. Additionally, we will apply the latest deep learning methodology that can efficiently learn large amounts of data.

## Figures and Tables

**Table 1:** Details of dataset.

Algorithm	$N_{\mathrm{tr}}$	$N_{\mathrm{val}}$	$N_{\mathrm{ts}}$	m(bit),l(bit)	Rounds
S-DES	55,000	30,000	15,000	8, 10	2
S-AES	900,000	500,000	200,000	16, 16	2
S-SPECK	10,000,000	2,000,000	100,000	32, 64	22

**Table 2:** Comparison with previous work (BAP and the number of parameters for S-DES).

Method	1st	2nd	3rd	4th	5th	6th	7th	8th	9th	10th	Parameters
[6]	0.64	0.74	0.71	0.58	0.64	0.8	0.54	0.6	0.84	0.8	805,930
This work (Res)	0.72	0.77	0.75	0.6	0.76	0.8	0.59	0.68	0.85	0.83	53,802
This work (Res+GLU)	0.72	0.79	0.77	0.62	0.75	0.81	0.59	0.66	0.87	0.85	55,092

Red font indicates high performance; Blue font indicates low performance; Gray background indicates this work.

**Table 3:** Bit accuracy probability by epoch for S-DES (V: vulnerable bit, S: safe bit).

Epoch	1st	2nd	3rd	4th	5th	6th	7th	8th	9th	10th
15	S	S		S	S	V	S	S	V	
25				S	S	V	S	S	V	
35				S	S	V	S	S	V	
95				S	S	V	S	S	V	
100				S		V	S		V	V

Red font indicates high performance; Blue font indicates low performance; Gray background indicates results are constant from 15-epoch to 100-epoch.

**Table 4:** BAP for S-AES (9∼12-bit key space).

Key	1st	2nd	3rd	4th	5th	6th	7th	8th	9th	10th	11st	12nd	13rd	14th	15th	16th
9-bit	-	-	-	-	-	-	-	0.7	0.7	0.69	0.69	0.7	0.69	0.69	0.7	0.69
10-bit	-	-	-	-	-	-	0.63	0.63	0.63	0.64	0.63	0.63	0.6	0.6	0.6	0.6
11-bit	-	-	-	-	-	0.52	0.53	0.52	0.53	0.52	0.52	0.53	0.52	0.51	0.52	0.52
12-bit	-	-	-	-	0.51	0.51	0.5	0.5	0.5	0.5	0.5	0.51	0.5	0.5	0.5	0.5

Gray background indicates completed results.

**Table 5:** BAP for S-SPECK (4∼6-bit key space).

Key	1st	2nd	3rd	4th	5th	6th
4-bit	-	-	0.54	0.53	0.53	0.53
5-bit	-	0.52	0.51	0.51	0.52	0.52
6-bit	0.51	0.51	0.51	0.51	0.50	0.51

Gray background indicates completed results.

## Data Availability

Not applicable.
